# Creative activity and accomplishment as indicators of polymathy among gifted and nongifted students

**DOI:** 10.3389/fpsyg.2023.1255508

**Published:** 2024-01-10

**Authors:** Ahmed M. Abdulla Alabbasi, Mark A. Runco, Alaa Eldin Ayoub

**Affiliations:** ^1^Department of Gifted Education, Arabian Gulf University, Manama, Bahrain; ^2^Creativity Research and Programming, Southern Oregon University, Ashland, OR, United States; ^3^Department of Educational Psychology, Aswan University, Aswan, Egypt

**Keywords:** polymathy, domain specificity, gifted students, creative activity and accomplishments, hierarchical regression analysis

## Abstract

This study examined domain specificity among 306 high-school students using the *Creative Activity and Accomplishment Checklist* (CAAC). The CAAC provides both the quantity of activity and quality of accomplishment scores, allowing an empirical test of possible polymathy among students, some of whom were gifted. Polymathy occurs when an individual performs creatively in more than one domain. This investigation’s two objectives were to replicate domain specificity studies with the newest version of the CAAC, which included new domains (i.e., technological and everyday creativity) and quality and quantity scores, and to use it to test for polymathy among students. Previous work with adults suggested that polymaths are creative in multiple domains. They often invest in creative avocations that support their professional creativity. Some evidence of polymathy was uncovered; however, it was not common in this sample. Support for domain specificity was reasonably clear in the present results, yet it was not all-or-nothing but rather a matter of degree. Domains overlapped to varying amount. The amount of overlap varied with the level of talent and from domain to domain. The clearest support for polymathy came from regression analyses which revealed a significant relationship between the quantity of activity in some domains and the quality of creative accomplishment in others. Limitations and future directions are discussed.

## 1 Introduction

Studies of creativity have advanced extensively over the past several decades (Runco and Abdullah, 2014). Initially, creativity was tied to the arts; for example, only artists were thought to be creative. Although this view persists in the form of an *art bias* (Cropley, 2014; Runco, 2014), it is widely held that creativity can be expressed in diverse domains (Gardner, 1983; Runco, 1986). The term domain has been defined as “the set of representations that underlie and support thinking in a specific area of knowledge” (Baer, 2011, p. 404). Evidence largely supports *domain specificity*, meaning that an individual who is creative in one domain is not necessarily creative in other domains.

Empirical demonstrations of domain specificity often use some form of a Creative Activity and Accomplishment Checklist (CAAC). The CAAC is highly praised; after a thorough review, Hocevar and Bachelor (1989) described it as the best method for measuring creativity. An advantage of the CAAC is that it provides domain-specific scores. Originally proposed by Chorness and Nottelmann (1957) and then extended and refined by Holland (1961) and Holland and Richards (1965), the CAAC has demonstrated good reliability in a variety of studies. Holland (1961) used the CAAC to demonstrate that extracurricular activity is not strongly related to academic success. Wallach and Wing (1969) used a CAAC in their research, showing that students’ school performance is not strongly related to creativity expressed when students are outside of school, which has been recently replicated by Runco et al. (2023) in a study that also used a version of the CAAC. Paek and Runco (2018) reported an ideational contribution to certain domains of creative activity. They also developed two new scales for the CAAC—one representing technological creativity and the other for everyday creativity. They also proposed a new scoring system to assess the quality of creative accomplishment. Paek et al. (2016) reviewed all research to date that has used some form of the CAAC.

One empirical investigation provided a modicum of validity for the CAAC (Runco et al., 1990). The self-ratings of students were significantly correlated with mothers’ reports of students’ creative activities and accomplishments. The students received one version of the CAAC, while the mothers received a parallel version, allowing them to describe the activities and accomplishments of their own children.

Previous studies with the CAAC have largely supported domain specificity; however, the newest form has new domains: technology and everyday creativity. Thus, it would make sense to replicate previous work with a new version of the CAAC. Additionally, newer versions provide both the quantity of activity and quality of accomplishment scores. Runco (1986) had a qualitative scoring method for the CAAC; however, it required judges and was thus open to subjectivity. Newer CAACs have an objective method of obtaining qualitative scores—another reason to replicate earlier work with a new version of the checklist.

The new method of qualitative scoring in the CAAC makes it possible to test for polymathy among students. Polymathy is an exception to domain-specific creativity. Polymathy occurs when an individual performs creatively in more than one domain. Root-Bernstein et al. (1995) reported that in a sample of Nobel Prize winners and members of the US National Academy of Science, the 11 individuals representing the highest level of eminence reported using their avocations in their professions. Twelve individuals in the same sample reported that the avocations were a waste of time. Thus, polymathy is not universal among eminent individuals. Another testable hypothesis is that creative polymathy is observed only in mature samples, which makes sense, given the benefits of avocations for professional efforts. Root-Bernstein and Root-Bernstein (2023) asked whether creative polymathy is found only among the eminent and questioned the need for the creative polymath to be productive. In this context, productivity appears to be a feature of professional life.

The CAAC can test for polymathy among students because it measures the quantity and quality of creative activity. The quantity of creative activities within a domain can be used as a proxy for what mature creators do avocationally. If it is an avocation, there is probably little social recognition, which is exactly what distinguishes the quality and quantity scores on the CAAC. The CAAC quality scores, contrastingly, can be used as a proxy for the professional work of mature creators, for here, there is social recognition. In sum, mature creators invest time in avocations, which sometimes actually contribute to the professional creativity that earns them social recognition; and here we tested this sort of thing by correlating the quantity of creative activity in one domain on the CAAC with the quality of creative accomplishment in other domains measured by the CAAC.

This investigation was not just a replication of the previous reports of domain specificity. It extends previous research by checking domain specificity with the CAAC, which includes new domains of creative activity. Additionally, we tested our conception of polymathy among students. Given the possibility that polymathy requires above-average talent, the sample for this research comprised both gifted and non-gifted students. In sum, this investigation was conducted to test for polymathy among students, compare gifted and nongifted students in this regard, and to determine statistically if what holds for mature creators (a relationship of quantity and quality of creative activity) is also apparent among adolescents.

## 2 Materials and methods

### 2.1 Participants and procedures

The current sample included 306 high-school students (*M_age_* = 15.76, SD *=* 2.05 years; 159 [52%] girls and 147 [48%] boys). Of these, 71 (23.2%) were classified as gifted based on the following assessments: (a) Screening Assessment for Gifted Elementary and Middle School Students (SAGES-2; Johnsen and Corn, 2001), (b) Creativity Assessment Packet (Williams, 1980), and (c) teachers’ nominations based on the Scales for Rating the Behavioral Characteristics of Superior Students (Renzulli et al., 2002). The study protocol was approved by the Directorate of Scientific Research, Ministry of Education, Bahrain. The link to the measures was sent to two private schools in Bahrain through official channels to obtain approval for the study’s instruments. This link was sent through the two schools to all high-school students (*N* = 685). A total of 322 responses were received; however, 16 were excluded because participants did not complete all assessments, resulting in 306 participants. Information about gifted students was obtained from the two schools. The nongifted students were not part of the gifted program because they did not meet the criteria for the gifted program. All participants were asked to provide their consent to participate on the first page of the survey. The instruments were administered online through SurveyMonkey.

### 2.2 Instruments

#### 2.2.1 CAAC

The CAAC has been used in many previous empirical studies on creativity (Holland and Richards, 1965; Wallach and Wing, 1969; Runco, 1986; Paek and Runco, 2017). The version used here sampled eight domains of creativity: (a) Writing (15 items), (b) Music (14 items), (c) Art (11 items), (d) Crafts (10 items), (e) Math and Science (6 items), (f) Technology (14 items), (g) Everyday Creativity (12 items), and (h) Sport (11 items). Four of the CAAC domains (Writing, Music, Art, and Everyday Creativity) included both quantity (e.g., “How often have you performed with a musical instrument?”) and quality items (e.g., “How often have you won an award in a musical competition?”), while the remainder (i.e., Crafts, Math and Science, Technology, and Sport) only included quantitative items. The CAAC in previous research had reliability ranging from 0.74 to 0.88 (Runco et al., 2017). It was also recently used with an Arab sample, and its reliability was above 0.90 (Runco et al., 2023). In the CAAC, each statement begins with “how often have you,” with four options ranging from *never* to *more than five times*. The questionnaire provided the following directions:

This inventory asks how often you have experienced various events. For each item, select the answer that best describes you and what you have done. Therefore, it is necessary to approximate. Remember: No names are used. Your responses are now confidential.

Here are the options:

1. You have never done an activity or accomplishment.
2. You have done this activity or accomplishment once or twice.
3. You have done this activity or accomplishment 3–5 times.
4. You have done this activity or accomplished it more than five times?

## 3 Results

The internal consistency was calculated for each CAAC domain. The reliability coefficients ranged from 0.73 to 0.91 (Table 1).

**Table 1:** Reliabilities coefficients of the study instrument.

Instrument	Reliability (alpha)
CAAC (Writing)	0.84
CAAC (Music)	0.84
CAAC (Art)	0.86
CAAC (Craft)	0.81
CAAC (Math & Science)	0.73
CAAC (Technology)	0.80
CAAC (Everyday Creativity)	0.77
CAAC (Sport)	0.91

CAAC, creative activity and accomplishment checklist.

### 3.1 Are students polymaths? If so, how many?

Polymathy is typically observed in eminent adult creators. Our approach was much more liberal because we were working with students. For example, we did not require that polymathic individuals contribute significantly to a profession. We were simply interested in the possibility that some students did work in one domain which was associated with their activity and achievement in another domain.

To determine how many gifted students displayed polymathy or, at least, a history of creativity in more than one domain, we calculated the 90-percentile score for each of the eight CAAC domains, allowing SPSS to create a new dichotomous variable (90% or above vs. below 90%). The “select cases” function was then used to determine the number of gifted and non-gifted students who scored 90% or above (*n* = 53; Table 2). As shown in Table 2, gifted students were overrepresented in all the CAAC domains (ranging from 55.9% to 92%).

**Table 2:** Representations of gifted and non-gifted students who scored 90% or above in all CAAC domains.

CAAC domain	Gifted *n*	Gifted percentage	Non-gifted *n*	Non-gifted percentage
Writing	17	56.7%	13	43.3%
Music	20	71.4%	8	28.6%
Art	23	79.3%	6	20.7%
Craft	20	74.1%	7	25.9%
Math & Science	19	63.3%	11	36.7%
Technology	17	70.8%	7	29.2%
Everyday creativity	23	92%	2	8%
Sport	19	55.9%	15	44.1%
**Total**	158		69	

CAAC, creative activity and accomplishment checklist.

Each of the 53 cases was individually traced to see if the same case (i.e., student ID) appeared in more than one domain to determine the number of gifted and non-gifted students who excelled in at least two domains. This could be considered polymathy, although there are caveats (e.g., reliance on the CAAC), which are outlined in the Discussion. Of the 53 students, 45 (84.9%) were gifted. Additionally, the results revealed the following: (a) 29 of the 53 participants (54.7%) scored higher than 90% in 2 domains (23 gifted and 6 non-gifted); (b) 8 participants (15.1%) scored higher than 90% in 3 domains (7 gifted and 1 non-gifted); (c) 7 participants (13.2%) scored higher than 90% in 4 domains (7 gifted and 0 non-gifted); (d) 3 participants (5.7%) scored higher than 90% in five domains (2 gifted and 1 non-gifted); 2 participants (3.8%) scored higher than 90% in 6 domains (2 gifted and 0 non-gifted); (e) 2 participants (3.8%) scored higher than 90% in 7 domains (2 gifted and 0 non-gifted); and (f) 2 participants (3.8%) scored higher than 90% in 8 domains (2 gifted and 0 non-gifted).

### 3.2 Regression analyses

One way to test for polymathy involved examining how the quantity of activity scores in any domain related to the quality of accomplishment scores in different domains. This parallels what has been observed in eminent creators: they often have an avocation where there is no social recognition but, at the same time, they are highly creative (and receive social recognition) professionally, in a different domain. Before comparing domains in this manner, it was reasonable first to determine whether the quantitative activity scores in any one domain predicted the quality of accomplishment scores in the same domain. To this end, two hierarchical regression analyses were performed. The decision for using this kind of regression was that interactions can only be tested with a hierarchical regression (Cohen, 1988). The first set of regression analyses used (a) the Total Quantitative CAAC score representing the quantitative items in the Writing, Music, Art, and Everyday Creativity domains and (b) the Total Qualitative score representing the qualitative items in the same four domains. This Total CAAC score was used to avoid conducting too many statistical analyses and to avoid Type 1 error. Moreover, total scores are often used in research using the CAAC (Elisondo et al., 2022; Runco et al., 2023). The Total Quantitative Score was centered based on Cohen et al. (2003) recommendation and entered in the first step of the regression, followed by Giftedness Status (gifted vs. non-gifted), and the Total Quantitative centered × Giftedness Status (that is, their interaction), each in a separate step of the regression analysis, while the Total Qualitative score was used as the dependent variable. This hierarchical testing of interactions was recommended by Cohen (1988). The hierarchical regression analyses indicated that the Total Quantitative CAAC score (Step 1) was significantly related to the Total Qualitative score (Adjusted *R*^2^ = 0.53, *β* = 0.73, *t* = 18.63, *p* < 0.001). Giftedness Status (Step 2; Δ*R*^2^ = −0.20, *β* = 0.57, *t* = 12.33, *p* < 0.001) and the interaction (Step 3; Δ*R*^2^ = 0.07, *β* = 0.63, *t* = 14.40, *p* < 0.001) were also significant.

The second set of regression analyses compared the quantitative scores in each domain with the qualitative scores within domains. As with the first set of analyses, Giftedness Status and the interaction were tested as separate steps in the regression analysis. Regarding the Writing domain, the Quantitative score was significantly related to the Qualitative score (Adjusted *R*^2^ = 0.34, *β* = 0.59, *t* = 12.65, *p* < 0.001). Giftedness Status (Δ*R*^2^ = −0.26, *β* = 0.29, *t* = 5.36, *p* < 0.001) and the interaction effect (Δ*R*^2^ = 0.07, *β* = 0.40, *t* = 7.59, *p* < 0.001) were also significantly related to the Quality of Writing. The Beta values of the final model show that the quantitative score in Writing was weighted more heavily in predicting the Qualitative Writing score than the Giftedness Status and the interaction. Gifted students scored higher than non-gifted students in Writing quality (see Table 3 for the means and standard deviations). In the Art domain, the quantitative score significantly predicted the quality of Art (Adjusted *R*^2^ = 0.31, *β* = 0.56, *t* = 11.86, *p* < 0.001). Giftedness Status (Δ*R*^2^ = −0.07, *β* = 0.50, *t* = 10.05, *p* < 0.001) and the interaction effect (Δ*R*^2^ = 0.07, *β* = 0.56, *t* = 11.95, *p* < 0.001) were also significant. Gifted students scored significantly higher than non-gifted students in Art (Table 3). A similar finding was obtained in the Music domain, in which the hierarchical regression analysis indicated that the quantitative score significantly predicted the quality of the Music scores (Adjusted *R*^2^ = 0.36, *β* = 0.60, *t* = 13.19, *p* < 0.001). Giftedness Status (Δ*R*^2^ = −0.26, *β* = 0.32, *t* = 5.86, *p* < 0.001) and the interaction effect (Δ*R*^2^ = 0.11, *β* = 0.46, *t* = 8.96, *p* < 0.001) were also significant. Again, gifted students outperformed non-gifted students concerning the quantity of creative musical activities. Finally, the results for the Everyday Creativity domain showed that the quantitative score (Adjusted *R*^2^ = 0.23, *β* = 0.48, *t* = 9.58, *p* < 0.001), Giftedness Status (Δ*R*^2^ = −0.04, *β* = 0.44, *t* = 8.57, *p* < 0.001), and the interaction effect (Δ*R*^2^ = −0.02, *β* = 0.42, *t* = 8.12, *p* < 0.001) were significantly related to the quality of Everyday Creativity. Table 4 presents the full regression models.

**Table 3:** Means and standard deviations for the gifted and non-gifted students.

Variables	Gifted (*n* = 71)		Non-gifted (*n* = 235)		*t*(304)	*p*	Cohen’s *d*
	*M*	SD	*M*	SD			
CAAC-Writing	2.34	0.505	1.79	0.470	8.54	< 0.001	1.157
CAAC-Music	2.08	0.705	1.45	0.428	9.19	< 0.001	1.245
CAAC-Art	2.86	0.598	1.96	0.577	11.29	< 0.001	1.530
CAAC-Crafts	2.25	0.551	1.55	0.428	11.10	< 0.001	1.504
CAAC-Math & Science	2.58	0.635	1.75	0.552	10.79	< 0.001	1.461
CAAC-Technology	2.27	0.439	1.68	0.420	10.25	< 0.001	1.388
CAAC-Everyday creativity	2.95	0.426	2.17	0.479	12.32	< 0.001	1.668
CAAC-Sports	1.88	0.791	1.29	0.451	7.93	< 0.001	1.074
CAAC-Writing quantity	2.74	0.594	2.15	0.578	9.06	< 0.001	1.227
CAAC-Writing quality	1.88	0.741	1.38	0.470	5.36	< 0.001	0.726
CAAC-Music quantity	2.16	0.741	1.48	0.443	9.38	< 0.001	1.271
CAAC-Music quality	2.21	1.064	1.50	0.681	5.86	< 0.001	0.794
CAAC-Art quantity	3.24	0.666	2.42	0.781	9.66	< 0.001	1.308
CAAC-Art quality	2.19	0.900	1.33	0.508	10.05	< 0.001	1.361
CAAC-Everyday quantity	3.03	0.521	2.29	0.556	11.21	< 0.001	1.519
CAAC-Everyday quality	2.67	0.635	2.03	0.696	8.57	< 0.001	1.161

A Bonferroni correction was applied to the 16 comparisons. It resulted in a significance level of (0.00312), which is higher than 0.001; thus, it is safe to conclude that the results presented in Table 3 are not influenced by Type I error. SD, standard deviation, CAAC, creative activity and accomplishment checklist.

**Table 4:** Hierarchical regression of associations between quantitative and qualitative CAAC scores within the same domain (*N* = 306).

Dependent variable	Variables	*β*	*SE*	*t*	*p*	95% CI
Writing (qualitative)	Writing (quantitative)	0.59	0.04	12.65	< 0.001	[0.446, 0.610]
	Giftedness status	0.29	0.07	5.36	< 0.001	[0.246, 0.531]
	Writing (quantitative) × Giftedness status	0.40	0.08	7.59	< 0.001	[0.480, 0.816]
Art (qualitative)	Art (quantitative)	0.56	0.04	11.86	< 0.001	[0.386, 0.540]
	Giftedness status	0.50	0.08	10.05	< 0.001	[0.643, 0.956]
	Art (quantitative) × Giftedness status	0.56	0.08	11.95	< 0.001	[0.770, 1.074]
Music (qualitative)	Music (quantitative)	0.60	0.06	13.19	< 0.001	[0.729, 0.984]
	Giftedness status	0.32	0.10	5.86	< 0.001	[0.404, 0.811]
	Music (quantitative) × Giftedness status	0.46	0.10	8.96	< 0.001	[0.723, 1.131]
Everyday creativity (qualitative)	Everyday creativity (quantitative)	0.48	0.06	9.58	< 0.001	[0.451, 0.685]
	Giftedness status	0.44	0.09	8.57	< 0.001	[0.584, 0.933]
	Everyday creativity (quantitative) × Giftedness status	0.42	0.11	8.12	< 0.001	[0.688, 1.129]

A Bonferroni correction was resulted in a significance level of 0.0125, which is higher than 0.001; thus, it is safe to conclude that the results presented in this table are not influenced by Type I error. CAAC, Creative Activity and Accomplishment Checklist.

These results indicate that practicing more activities in one domain is related to higher achievement within the same domain. The relationship varied between the gifted and non-gifted samples. The next set of analyses examined whether practicing more activities (quantity) in some domains (i.e., Math and Science, Technology, Crafts, and Sport) predicted the quality of creative activities in other domains (i.e., Writing, Art, Music, and Everyday Creativity), speaking directly to the question of polymathy among gifted and non-gifted students.

First, regarding the Math and Science domain of the CAAC, Step 1 (with an average score across Math and Science activities) was significantly related to a Total Qualitative score representing Writing, Art, Music, and Everyday Creativity (Adjusted *R*^2^ = 0.12, *β* = 0.35, *t* = 6.54, *p* < 0.001). Giftedness Status (Step 2; Δ*R*^2^ = 0.21, *β* = 0.58, *t* = 12.33, *p* < 0.001) and the Math/Science interaction with Giftedness Status (Step 3; Δ*R*^2^ = −0.18, *β* = 0.39, *t* = 7.50, *p* < 0.001) were significantly related to the Total Qualitative score.

The same kind of analysis was repeated using the Technology domain to predict the Total CAAC Qualitative score. The Technology domain (Adjusted *R*^2^ = 0.21, *β* = 0.47, *t* = 9.18, *p* < 0.001), Giftedness Status (Δ*R*^2^ = 0.12, *β* = 0.58, *t* = 12.33, *p* < 0.001), and the Technology × Giftedness Status (Δ*R*^2^ = −0.09, *β* = 0.48, *t* = 9.67, *p* < 0.001) significantly predicted the Total Quality score. Similar findings were found for the Crafts domain, where Step 1 (Adjusted *R*^2^ = 0.27, *β* = 0.52, *t* = 10.71, *p* < 0.001), Step 2 (Δ*R*^2^ = 0.06, β = 0.58, *t* = 12.33, *p* < 0.001), and Step 3 (Δ*R*^2^ = −0.04, *β* = 0.54, *t* = 11.11, *p* < 0.001) significantly predicted the Total Qualitative score. Finally, regarding the Sport domain, Step 1 (Adjusted *R*^2^ = 0.07, *β* = 0.28, *t* = 5.03, *p* < 0.001), Step 2 (Δ*R*^2^ = 0.256, *β* = 0.58, *t* = 12.33, *p* < 0.001), and Step 3 (Δ*R*^2^ = −0.251, *β* = 0.29, *t* = 5.22, *p* < 0.001) were significantly related to the Total Quality score. Table 5 presents the full regression models. Bivariate correlational analyses are presented in Tables 6, 7.

**Table 5:** Hierarchical regression of associations between quantitative domains of the CAAC and the total qualitative score.

Dependent variable	Variables	*β*	SE	*t*	*p*	95% CI
Total qualitative score	Math & Science	0.35	0.04	6.54	< 0.001	[0.171, 0.318]
	Giftedness Status	0.58	0.05	12.33	< 0.001	[0.537, 0.740]
	Math & Science × Giftedness status	0.39	0.06	7.50	< 0.001	[0.334, 0.572]
	Technology	0.47	0.05	9.18	< 0.001	[0.348, 0.538]
	Giftedness status	0.58	0.05	12.33	< 0.001	[0.537, 0.740]
	Technology × Giftedness status	0.48	0.08	9.67	< 0.001	[0.634, 0.958]
	Crafts	0.52	0.04	10.71	< 0.001	[0.367, 0.532]
	Giftedness status	0.58	0.05	12.33	< 0.001	[0.537, 0.740]
	Crafts × Giftedness status	0.54	0.06	11.11	< 0.001	[0.597, 0.854]
	Sport	0.28	0.04	5.03	< 0.001	[0.131, 0.300]
	Giftedness status	0.58	0.05	12.33	< 0.001	[0.537, 0.740]
	Sport × Giftedness status	0.29	0.06	5.22	< 0.001	[0.197, 0.435]

The total qualitative score represents qualitative items in the writing, art, music, and everyday creativity domains. CAAC, creative activity and accomplishment checklist.

**Table 6:** Correlation between gifted (*n* = 71) and non-gifted (*n* = 235) Samples in the quantitative CAAC scores.

	Writing	Music	Art	Craft	Math & Science	Technology	Everyday Creativity	Sport
Writing		0.413**	0.419**	0.439**	0.273*	0.418**	0.385**	0.072
Music	0.490**		0.353**	0.434**	0.289*	0.218	0.396**	0.198
Art	0.441**	0.435**		0.665**	0.263*	0.466**	0.545**	0.085
Craft	0.320**	0.316**	0.535**		0.314**	0.463**	0.710**	0.161
Math & Science	0.293**	0.317**	0.295*	0.333**		0.325**	0.328**	0.217
Technology	0.425**	0.377**	0.372**	0.305**	0.517**		0.525**	0.244*
Everyday creativity	0.378**	0.347**	0.483**	0.606**	0.503**	0.500**		0.341**
Sport	0.196**	0.283**	0.200**	0.265**	0.464**	0.338*	0.299**	

The results for the gifted sample (*n* = 71) are shown above the diagonal, and the results for the non-gifted sample (*n* = 235) are shown below the diagonal. CAAC, creative activity and accomplishment checklist. *p < 0.05. **p < 0.01.

**Table 7:** Correlation between different CAAC quantitative domains for the total sample (*N* = 306).

	Writing	Music	Art	Craft	Math & Science	Technology	Everyday Creativity	Sport
Writing		0.476**	0.435**	0.345**	0.289*	0.421**	0.378**	0.172**
Music			0.417**	0.345**	0.313**	0.343**	0.359**	0.264**
Art				0.571**	0.293**	0.404**	0.502**	0.169**
Craft					0.331**	0.350**	0.635**	0.237**
Math & Science						0.477**	0.463**	0.408**
Technology							0.510**	0.305**
Everyday creativity								0.301**

CAAC, creative activity and accomplishment checklist. *p < 0.05. **p < 0.01.

## 4 Discussion

There are several notable results of the analyses. The correlations in Table 6, for example, largely suggest domain specificity. The highest bivariate correlation among domains in the gifted sample was 0.71, and the highest in the non-gifted sample was 0.61. The former indicates that 50% of the variance in the domain scores was shared, while the latter indicates that 37% of the variance was shared. Notably, the highest correlation was between Crafts and Everyday Creativity; two domains where overlap is expected. Some domains were negligibly correlated (e.g., 0.072 and 0.085). Of course, domain specificity is not all-or-nothing but a matter of degrees.

The percentage results (Table 2) also mostly supported domain specificity. Only 29 of the 306 students (90% or above) excelled in two or more domains. These percentage results, as well as the regression analysis results, indicate that the degree that various domains are correlated tends to depend on giftedness status. One tentative conclusion is that domain specificity is the norm; however, some polymaths do well in more than one domain (cf. Table 8).

**Table 8:** Number of gifted and non-gifted students who excelled in two or more Domains.

Number of domains above 90%	Students *n* (gifted)	Students *n* (non-gifted)
Two domains	23	7
Three domains	7	1
Four domains	7	0
Five domains	2	1
Six domains	2	0
Seven domains	2	0
Eight domains	2	0
**Total**	45	9
**Percentage polymathic**	63.4%	3.83%

The regression analyses using the separate domains, with the quantitative CAAC scores as predictors and quality of creative accomplishment as criteria, were consistent with Simonton’s (2003) constant probability of success model. Briefly, the logic here is that the more one does (quantity), the more likely it is that something of high quality will be discovered. If students write 100 poems (Writing domain), *ceteris paribus*, they are more likely to produce an award-winning poem than those who write only one.

The results of the last set of regression analyses indicate that participating in creative activities, even in other domains, can predict qualitative achievement (social recognition) in different domains. This may be the clearest form of support for polymathy. Recall here that polymathy in eminent samples is apparent when the individual has some creative avocation but also performs at an excellent level in a different domain—Einstein’s practice of the violin while changing the field of physics can be cited here. In this investigation, working in some avocations with no professional expectations was operationalized concerning the quantity of activity. Professional achievement, meanwhile, is more strongly related to the quality of one’s creative accomplishments.

This study used the CAAC to assess the quality and quantity of creative performance. The CAAC has been used for many years (e.g., Holland and Richards, 1965; Wallach and Wing, 1969; Paek and Runco, 2018) and is highly respected (Hocevar and Bachelor, 1989). Still, it is a self-report; thus, it is open to certain biases (e.g., memory and honesty). One study reported correlations between children’s CAAC reports and those of their mothers, implying a mild form of validation (Runco et al., 1990). In this investigation, reliabilities were quite good. Future research could extend this line of work by testing other domains or perhaps by adding controls for the limitations such as memory and honesty. Runco et al. (2014) developed a lie scale for the Runco Ideational Behavior Scale, and a similar scale can be written into the CAAC. The variance attributed to dishonesty could then be controlled, and polymathy analyzed. Future research might examine polymathy among students with a version of the CAAC that only includes domains which have been found in adult polymaths, for more focus, but with more questions within each domain for more information. Future research should certainly replicate this study using other samples. The present sample was homogeneous and perhaps not informative about countries and cultures other than the one sampled. Further, the present investigation had imbalanced group sizes, and although hierarchical regression is not sensitive to this sort of thing, it could be avoided if there is a replication or extension. Additional research might also collect data to determine why certain domains were associated with one another (e.g., technology and writing) and what, if anything, in participants’ developmental histories contributed to polymathy. Additional CAAC domains might be examined in the future, as well.

There are varying kinds of polymathy and different approaches to studying it. Some research focused on skill, ability, knowledge, or worldviews (e.g., Sriraman, 2009; Araki, 2018). Future research could explore if these variables help explain the differences (gifted vs. non-gifted) and associations among the domains uncovered here. Additionally, Root-Bernstein and Root-Bernstein (2020a) described a kind of intra-domain polymathy that bridges (rather than separates) domains. Indeed, quite a few eminent individuals are famous for their work within one domain, but in actuality, their thinking is borrowed from more than one domain (Gardner and Wolf, 1988). Such individuals are polymaths in their thinking but not in their performance. That being said, polymathy is not always constrained by domain boundaries. The research reported here only examined trans-domain polymathy. Root-Bernstein and Root-Bernstein (2020b, p. 377) distinctions between limited, proper, serial, and passive polymaths, one key question for us was, are (trans-domain proper) polymaths only found among eminent samples, or do we see them among gifted students? Based on the data reported here, the answer is that this particular kind of polymath is apparent among gifted students.

## Data availability statement

The raw data supporting the conclusions of this article will be made available by the authors, without undue reservation.

## Ethics statement

The studies involving humans were approved by Directorate of Scientific Research, Ministry of Education, Bahrain. The studies were conducted in accordance with the local legislation and institutional requirements. Written informed consent for participation in this study was provided by the participants’ legal guardians/next of kin.

## Author contributions

AMA: Methodology, Software, Validation, Writing – review & editing. MAR: Conceptualization, Methodology, Project administration, Writing – original draft. AEA: Data curation, Formal analysis, Validation, Writing – review & editing.
